# SCA8 CAG/CTG Expansions, a Tale of Two *TOXIC*ities: A Unique or Common Case?

**DOI:** 10.1371/journal.pgen.1000593

**Published:** 2009-08-14

**Authors:** Karine Merienne, Yvon Trottier

**Affiliations:** Department of Neurobiology and Genetics, Institute of Genetics and Molecular and Cellular Biology (IGBMC), UMR 7104-CNRS/INSERM/UdS, BP10142, Illkirch, CU de Strasbourg, France; The Hospital for Sick Children and University of Toronto, Canada

A dozen dominant genetic disorders are caused by aberrant expansions of CAG or CTG trinucleotide repeats. As these repeats are complementary sequences, the expansions are incurred by both strands. However, it is the genic or transcribed strand that has defined the CAG and CTG repeat diseases. CAG repeat–associated diseases represent a group of neurological disorders, including Huntington's disease, dentatorubral pallidoluysian atrophy, spinal and bulbar muscular atrophy, and several spinocerebellar ataxias (SCA1–3, 6, 7, and 17), which are all caused by an expansion of CAG repeats in the coding region of genes with otherwise no other common features. The resulting mutant, toxic proteins bear a polyglutamine expansion (polyQ), which is sufficient to trigger a neuronal pathology [1]. The propensity of polyQ-expanded proteins to progressively misfold and aggregate appears to be doubly deleterious. First, it impairs normal functions or turn-over of host proteins. Second, polyQ aggregates alter neuronal physiology by activating stress responses or recruiting essential components of the cellular machinery [2]. In contrast, the CTG repeat expansion responsible for myotonic dystrophy type 1 (DM1), a multi-systemic disease affecting mainly the muscle but also the brain, is located in the 3′-untranslated region of the *DMPK* gene. The toxic entity is the CUG-expansion transcript, which adopts secondary structures and forms RNA foci, thereby sequestering RNA-binding proteins implicated in pre-mRNA splicing [3]. Muscleblind-like (MBNL) proteins are splicing factors whose recruitment to RNA foci leads to misregulated splicing of tissue-specific targets. In addition, CELF proteins, including CUG binding protein 1 (CUGBP1), are up-regulated in DM1 tissues. Both events contribute to the reversal from adult to fetal or developmental splicing patterns [4]. Thus, so far, it has been widely accepted that toxic RNA gain-of-function and toxic protein gain-of-function mechanisms underlie the distinction between CTG and CAG repeat–associated diseases [5]. Two articles on spinocerebellar ataxia type 8 (SCA8) published by the group of Laura Ranum, first in 2006 [6] and now in this issue of *PLoS Genetics* [7], suggest that this view is simplistic, deserving of attention and revision.

Since its discovery, SCA8 has been a curiosity among triplet repeat disorders. It was the first dominant SCA apparently not caused by a CAG expansion–encoding polyglutamine tract, as the uncovered mutation was a CTG repeat expansion in a transcribed gene (*ATXN8OS*) lacking open reading frames (ORFs) [8]. In 2006, Ranum and colleagues [6] designed transgenic mice carrying a bacterial artificial chromosome (BAC) encompassing the entire human SCA8 locus, with a normal or expanded CTG allele. Characterization of the model revealed bidirectional expression of both CUG- and CAG-expansion transcripts. Unexpectedly, the authors found that the CAG transcript was translated into a nearly pure polyQ protein that formed typical neuronal nuclear inclusions, the specific hallmark of polyQ diseases. The data suggested, therefore, that the pathology resulted, in part, from a toxic protein gain-of-function mechanism and raised the possibility that SCA8 could be caused by both toxic protein and toxic RNA gain-of-functions.

Daughters et al. now present a body of evidence suggesting that the toxic RNA pathomechanism substantially contributes to SCA8 pathology [7]. The authors show that the CUG-repeat transcripts expressed from the SCA8 locus form RNA foci in specific neurons in SCA8 patient and mouse brains. In addition, MBNL1 protein colocalizes with these RNA foci. Furthermore, the authors provide evidence for a genetic interaction between SCA8 and *Mbnl1* gene loci by showing that loss of the *Mbnl1* gene in SCA8 BAC^exp^ mice increases the motor phenotype. These results extend to mice earlier observations in flies showing that SCA8 CUG transcript expression caused toxicity that can be modulated by mutation in *muscle-blind* or three other genes encoding RNA-binding proteins [9]. Importantly, using cross-linking and immunoprecipitation (CLIP) analysis with an anti-CUGBP1 antibody, Ranum and colleagues identify a specific CUGBP1 RNA target, the *GABA-A transporter 4* (*GAT4*) transcript, which shows up-regulation and a shift in alternative splicing favoring exon 7 insertion in SCA8 mouse and patient brains, resembling expression features detected in the fetal cortex. Interestingly, these changes are specific to SCA8 patients, as *GAT4* expression and splicing pattern are normal in DM1 brain. Finally, up-regulation of the *GAT4* gene product, which is primarily detected in the granular cell layer of the cerebellar cortex in the SCA8 mouse, fits well with the increased physiological response of granular cells to mossy fiber inputs. The authors hypothesize that transcript-skipping exon 7 is targeted for nonsense-mediated decay, and that shifting in exon 7 insertion in SCA8 patients underlies *GAT4* up-regulation. Together, these data indicate that the CUG-expansion transcript expressed at the SCA8 locus is indeed toxic.

Thus, both studies from Ranum's group provide the proof of concept that toxic RNA and toxic protein gain-of-function mechanisms can, together, be involved in SCA8, although the relative contribution of each mechanism remains to be determined. The next obvious question to address is whether bidirectional expression of CAG- and CUG-expansion transcripts is a general feature of CAG/CTG diseases and whether the combined toxic effects of RNA and protein gains-of-function could underlie the pathomechanisms involved in these diseases and account for their complexities. Bidirectional expression at the DM1 locus has been reported, but the antisense transcript does not appear to be translated into a polyQ protein [10]. More puzzling is the case of Huntington's disease–like 2 (HDL2), a dominant inherited disease closely resembling Huntington's disease and caused by CAG/CTG repeat expansion in the *JPH3* gene [11]. While expression of CUG-expansion *JPH3* transcripts forming toxic RNA foci was demonstrated, the presence of polyQ aggregates was highly suggested by immunodetection using an antibody specifically detecting polyQ expansions, i.e., the 1C2 antibody [12],[13]. If it happens that expression of antisense CUG-expansion transcripts is common in polyQ disorders, undoubtedly this will open new routes of investigation of their pathomechanism. Specifically, such a discovery could help to solve the issue of tissue selectivity, which remains puzzling. Indeed, the pattern of affected tissues correlates neither with the pattern of expression of CAG-expansion transcripts nor with the pattern of polyQ neuronal inclusions. In addition, degenerating neurons differ between polyQ diseases. For instance, the striatum preferentially degenerates in Huntington's disease, while the cerebellum is mostly affected in SCAs. The possible existence of antisense CUG-expansion transcripts could underlie some aspects of tissue selectivity. Toxic RNA effects could add to disease pathomechanism complexity. The relative contribution of both mechanisms (toxic RNA versus toxic protein) could also be tissue- or cell-specific, depending on the relative expression level of CAG- and CUG-expansion transcripts. Finally, data obtained from SCA3 *Drosophila* models suggested that CAG-expansion transcripts could also be toxic [14]. In that case, the toxicity threshold appeared to exceed 200 CAG repeats, which represent very rare disease alleles in polyQ disorders. One should, however, keep in mind that such high repeat numbers might be reached in some brain regions as a consequence of somatic repeat instability, as is illustrated in the case of Huntington's disease [15]. However, while toxic CUG diseases are typically thought to be associated with large expansions, in the SCA8 patients and mice studied by Ranum, the expansions were relatively short—within the range of many polyQ diseases. Thus, the potential connection between the RNA world and polyQ diseases now has to be clarified.

**Figure 1:**
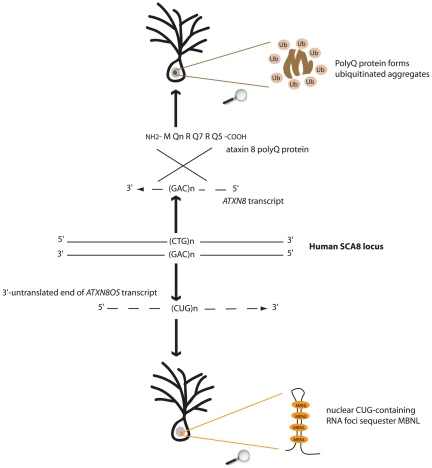
Both toxic-protein (polyQ) and toxic-RNA gain-of-functions might underlie SCA8 pathogenesis. Bidirectional expression of CAG- and CUG-transcripts at the human SCA8 locus results in production of a polyQ protein forming ubiquitinated aggregates and accumulation of RNA foci sequestering MBNL splicing factor.
